# Charge Carrier Dynamics at the Perovskite Interface with Self-Assembled Monolayers

**DOI:** 10.1021/acsami.4c10223

**Published:** 2024-10-18

**Authors:** Ernestas Kasparavičius, Marius Franckevičius, Simonas Driukas, Vidmantas Gulbinas

**Affiliations:** Center for Physical Sciences and Technology, Saulėtekio av.3, Vilnius 10257, Lithuania

**Keywords:** perovskite, self-assembling monolayers, hole transport layer, photocurrent dynamics, photovoltage transients, fluorescence, ion redistribution

## Abstract

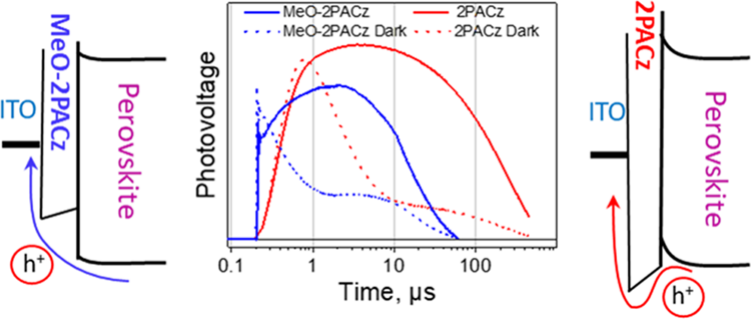

Self-assembled monolayers (SAMs) deposited on the hole-collecting electrodes of p–i–n perovskite solar cells effectively replace bulky hole transporting layers. However, the mechanism by which monolayers control the electronic processes and how they depend on the properties of the monolayer molecules remain poorly understood. In this study, we developed a simplified perovskite solar cell imitator with blocked electron extraction to investigate the photocurrent dynamics between the perovskite and the hole-collecting ITO electrode. We investigated the photoluminescence and photovoltage dynamics under short laser pulse excitation and addressed the influence of bulky and monomolecular hole transport layers. Our findings reveal that the photovoltage dynamics is significantly affected by the properties of the transport and perovskite layers, which in turn depend on the methods of sample preparation and exploration. Photocurrent dynamics is determined by several processes, including charge carrier displacement in the local electric field, hole transport to ITO, trapping of holes in interface trap states, and electron–hole recombination at the interface. We propose a model that takes into account molecular dipole moments and their ionization potentials to partially explain the different influences of different monolayers on the hole extraction and interfacial recombination rates. Additionally, the photovoltage dynamics also strongly depends on the illumination of the sample and shows memory effects that persist over minutes and hours and are attributed to the redistribution of ions.

## Introduction

Solar cell technology plays a substantial role in the global energy landscape, potentially evolving into a major energy source.^1^ Achieving this objective, however, necessitates the development of innovative solar cell technologies.^2^ Over the past decade, notable advancements have been achieved in enhancing the performance of perovskite solar cells (PSCs).^3^ Currently, the efficiency of PSCs has reached a record of 26.15%;^4^ however, several obstacles still hinder their commercialization, in particular their stability and reproducibility.^5^

Perovskite solar cells consist of several layers, each made of different materials, which play a crucial role in determining overall PSC performance.^6^ One particularly essential component is the hole transporting layer, which enables efficient extraction of holes from the perovskite, blocks extraction of electrons, and helps in preventing unwanted recombination.^7^ In 2018, a novel class of hole transporting materials (HTMs), self-assembling monolayers (SAMs), was introduced, demonstrating promising performance in p–i–n-type perovskite solar cells.^8^ Compared with the commonly used PTAA, these SAMs facilitate superior efficiency and stability, key factors for achieving the high performance of p–i–n PSCs.^9^ It should be noted that SAMs as interlayers between photoactive materials and electrodes were also widely used in organic,^10−12^ quantum dot,^13^ or dye-sensitized^14^ solar cells and in other electronic devices.^15^ SAMs function as charge-selective contacts and offer several additional advantageous properties: they require minimal material quantities and demonstrate compatibility with various substrates, including transparent conductive oxides serving as one of the electrodes. They can be deposited via spin-coating,^9^ by dipping the substrate into the solution,^16^ or by vapor-phase deposition.^17,18^ SAMs ensure high reproducibility, excellent surface contact with perovskite, and easy and simple fabrication.^19−22^ Although significant progress has been made toward achieving high efficiency,^23^ the influence of various SAMs on the performance of perovskite solar cells has not been sufficiently explored. The application of SAMs in perovskite solar cells has been discussed in several recent reviews.^6,24−^ Newly synthesized SAMs, composed of diverse carbazole-based molecules, have demonstrated promising results. However, there remains a lack of knowledge and comprehensive explanations concerning the dynamics of charge carriers at the SAM–perovskite interface.^27^ The electrode modification by SAM molecules typically alters several critical parameters. For example, the work function of ITO can be tuned through chemical modification of its surface, as demonstrated in previous studies.^28−30^ Additionally, changes in Schottky energy^31^ and wettability significantly impact the morphology of the deposited perovskite film. The thickness of the monolayer^15^ also plays a crucial role, affecting the charge transport distance and rate. Furthermore, the spacer group controls charge transport at the interface, thereby influencing the electronic conductivity and optical properties of the PSCs^32^

Several hypotheses have been proposed to explain the primary role of SAMs in influencing device performance.^33^ In addition to selective transmittance for different types of charge carriers, electric dipoles of SAM interlayers were suggested to change the apparent work functions of electrodes and facilitate charge extraction by bending the energy bands of perovskite.^25^ Das et al. recently experimentally investigated band bending and demonstrated its importance for the performance of perovskite solar cells.^34^ Yamaguchi et al. used photoelectron yield and electron spin resonance measurements together with DFT calculations to address the modification of the electrode work function by a 2PACz monolayer.^35^ SAMs also have the potential to serve as deactivators of recombination centers.^36,37^ Levine et al. showed that transient surface photovoltage (SPV) and transient photoluminescence provide complementary information about charge transfer kinetics and trapping/detrapping mechanisms at the SAM-modified ITO/perovskite interface.^36^ The study of photovoltaic parameters revealed that the specific characteristics of different moieties in carbazole-based compounds played a crucial role in PCE performance.^38^ By incorporation of an extended π-conjugation system between the carbazole core and the terminal group, an energetically compatible interface with the perovskite absorber was achieved. This system enhances hole transport, blocks electrons effectively, and passivates the perovskite surface, reducing energy losses.^38^ Electrochemical impedance spectroscopy (EIS) was also used for the quantitative analysis of charge transfer resistance in PSCs.^39^ Shih et al. demonstrated a decrease of the interfacial charge transfer resistance following modification of TiO_2_ by an amino acid SAM that aligned with the orientation of perovskite crystal grains, suggesting a strong correlation between charge transfer resistance and perovskite crystal orientation.^40^ The improved intermolecular charge transfer properties of SAMs greatly influence carrier dynamics at the perovskite/HTM interface in PSCs. Consequently, the rapid hole extraction and transfer processes effectively reduce charge accumulation and electron recombination at this interface.^37^

Nevertheless, the mechanism of charge transport through SAMs in perovskite solar cells is still the subject of ongoing debate. Despite many advancements, a significant gap exists in understanding the mechanisms governing charge selectivity in SAMs and the precise pathways of charge transport when perovskite solar cells are exposed to light.

In this work, we investigate photovoltage and photocurrent transients in simplified solar cell samples together with fluorescence decays to unveil charge carrier dynamics at the hole transport layer and perovskite interface. The most popular and widely utilized SAMs, which currently attract the greatest interest, are selected^41,42^ These techniques offer insights into carrier transport, accumulation, recombination, and ion motion.^43,44^ We demonstrate that the carrier transport dynamics depends on the ionization energies and dipole moments of monolayer molecules and also in a complex way on the properties of the perovskite layer, which are crucially affected by ion motion initiated by device illumination.

## Materials and Methods

### Materials

Patterned ITO glass substrates (150 mm × 200 mm, 20 Ω per square, 8 pixels) and PTAA for perovskite applications were from Ossila. PbI_2_, PbBr_2_ 2PACz, Me-4PACz, MeO-2PACz, and Br-2PACz were from TCI Chemicals. Dimethyl sulfoxide (DMSO), chlorobenzene, anisole, poly(3,4-ethylenedioxythiophene) (PEDOT:PSS), polystyrene, cesium iodide, and dimethylformamide (DMF) were purchased from Sigma-Aldrich. Methylammonium bromide (MABr) and formamidinium iodide (FAI) were ordered from Greatcell Solar Materials. All of the commercial materials were used as received.

### Perovskite Film Preparation

The perovskite films were prepared according to a method reported by Saliba et al.^45,46^ CsMAFA-based (Cs_0.05_(MA_0.17_FA_0.83_)_0.95_Pb(I_0.83_Br_0.17_)_3_) perovskite films were prepared according to the literature.^45,46^ The perovskite solution was spin-coated in a one-step program at 4000 rpm with an acceleration of 800 rpm/s for 35 s in a nitrogen atmosphere glovebox. During the program, 250 μL of anisole was poured onto the spinning substrate 10 s prior to the end of the program. Films with Cs-containing perovskites turned dark immediately after spin-coating. The films were then annealed at 100 °C for 30 min in a nitrogen atmosphere.

### Device Fabrication

The samples used for the investigation of the hole transport processes were prepared on glass substrates, each with 8 patterned ITO electrodes. The patterned ITO substrates were ultrasonically cleaned separately in a 2% Hellmanex aqueous solution, deionized water, acetone, and isopropanol for 15 min each. After drying under nitrogen flow, the substrates were further cleaned using plasma for 10 min. Subsequently, SAM layers were deposited according to the procedure suggested in ref (9). SAM molecules were dissolved in 2-propanol at a concentration of 1 mmol/L and spin-coated at 3000 rpm. After spin-coating, the substrates were heated at 100 °C for 10 min in a nitrogen atmosphere glovebox. A PEDOT:PSS layer was spin-coated at 4000 rpm from 2% solution in H_2_O and subsequently heated at 100 °C for 10 min. PTTA was spin-coated from a chlorobenzene solution at a concentration of 8 mg/mL at 4000 rpm. Next, the perovskite layer was deposited as described above. The samples were completed by spin-coating an insulating polystyrene layer from a chlorobenzene solution with a concentration of 10 mg/mL at 4000 rpm and finally an upper silver electrode formed by vapor deposition.

**Figure 1:**
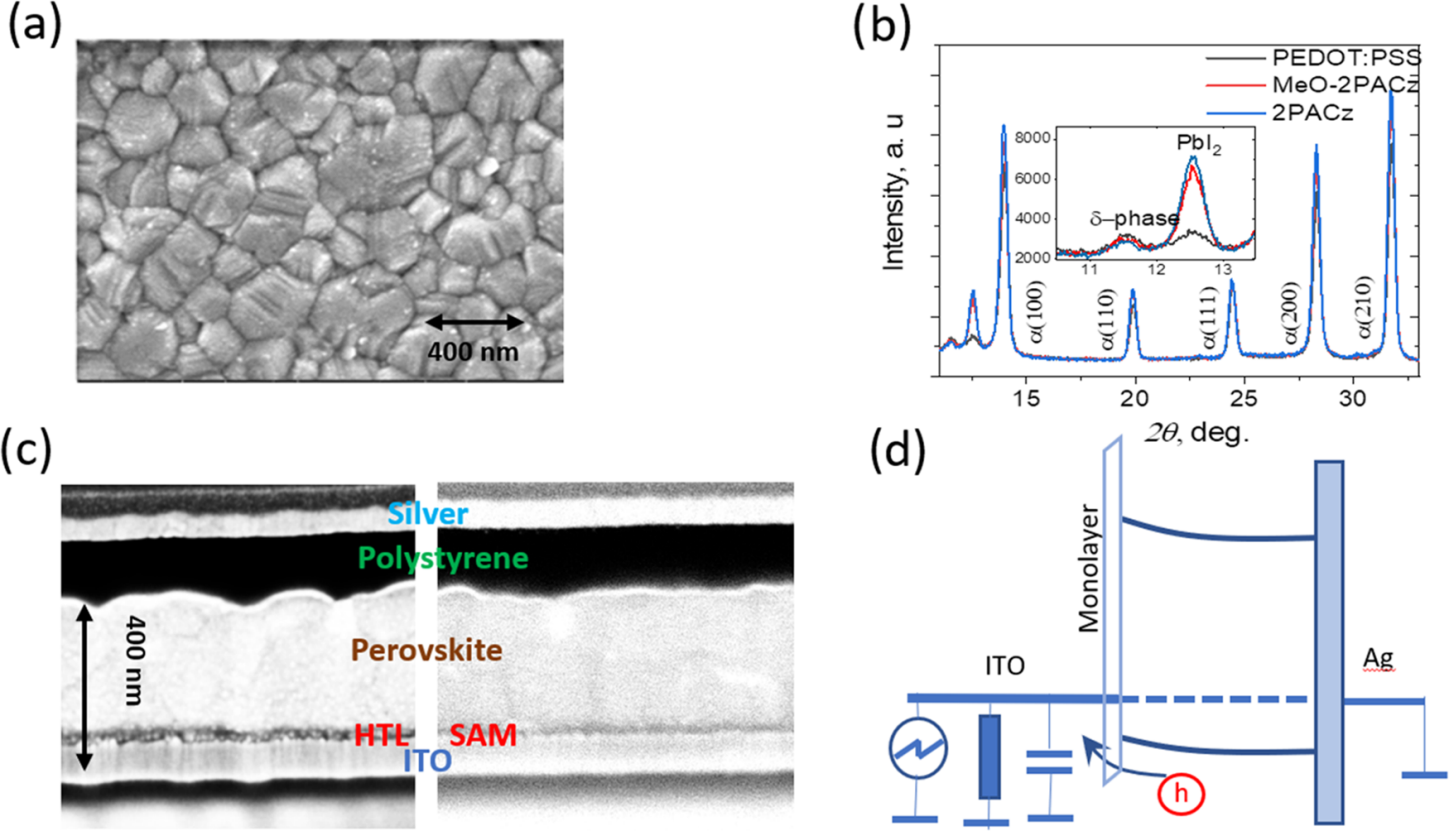
(a) Surface scanning electron microscopy image of perovskite. (b) XRD spectra of perovskite deposited on different transport layers. (c) Cross-sectional SEM images of perovskite samples formed on PEDOT:PSS and 2PACz SAM. (d) Schematic representation of the sample in energy levels and electrical scheme for photoresponse measurements.

We used the names of the transport layers to name the samples. To minimize accidental scattering of the sample properties, several 8-electrode samples with each transport layer were prepared and investigated. Figure 1a shows a surface SEM image of the perovskite layer on ITO. Surface SEM images of layers on ITO with other polymeric and SAM transport layers are presented in the Supporting Information (SI). The perovskite layers were composed of 200–400 nm grains. X-ray diffraction (XRD) spectra of the perovskite layers presented in Figure 1b show typical perovskite spectra but reveal the presence of some residual PbI_2_. Both SEM images and XRD spectra show no clear difference in the crystallographic structures of perovskites deposited on different transport layers. As described above, the samples were prepared in a similar way as inverted p–i–n solar cells, except that an insulating polystyrene layer was deposited on the perovskite instead of the electron transport layer. This modification blocked the electron extraction of the metal electrode, which simplified the interpretation of the photocurrent results and allowed easier investigation of carrier extraction, trapping, and recombination at the bottom interface. Figure 1c shows a cross-sectional SEM image of a perovskite sample formed on PEDOT:PSS and 2PACz SAM. PEDOT:PSS forms a layer with a thickness of tens of nanometers, while the SAM layer is much thinner and hardly resolved in the SEM image. The polystyrene layer forms a capacitor that is connected in series with the incomplete perovskite solar cell. It blocks the continuous current but transmits photocurrent transients and, thus, enables the observation of the photocurrent transients generated by optical pulses and monitoring of the motion of holes and electrons within the perovskite layer and their transfer to the hole transporting layer or to the ITO electrode.

### Experimental Measurements

Investigation of the transient photoresponse was performed in two measurement regimes: in the photovoltage regime using 1 MΩ oscilloscope input and in the transient photocurrent regime using 50 Ω input. Using 50 Ω input, we have directly measured the photocurrent. However, the time resolution was limited by the RC time of about 0.3 μs determined by oscilloscope resistance and the sample capacitance of about 6 nF. In this regime, we were able to apply voltage to the incomplete solar cell by using rectangular electrical pulses, which were transmitted through the polystyrene-formed capacitance. However, the voltage value was determined by thicknesses and dielectric constants of perovskite polystyrene and hole transport layers and was difficult to estimate, mainly because of the strongly time-dependent dielectric constant of perovskite.^47^ Measurements with a 1 MΩ resistance provided integrated current (photovoltage) kinetics. Here, the sample capacitance served as an integrating capacitance with a discharging time constant of about 6 ms. This measurement regime has no limitation of the resolution time related to the sample capacitance and thus enables time resolution of several nanoseconds, but the maximal photocurrent observation time was limited below 6 ms. On the other hand, the voltage drop across the perovskite layer was negligible in the case of measurements in the integrated photocurrent mode because the resistance of the perovskite layer was much lower than 1 MΩ. Therefore, such measurements were performed at zero applied external voltage. When the sample was optically excited, photogenerated charge carriers were extracted to the ITO electrode connected to the oscilloscope, detecting the integrated photocurrent. Thus, the positive voltage corresponds to hole extraction to ITO.

Time-resolved photoluminescence measurements were performed by means of the streak camera Hamamatsu C5680 coupled with a femtosecond Yb:KGW oscillator (Light Conversion Ltd.) generating 80 fs 1030 nm pulses at a 76 MHz repetition rate. The oscillator pulses were frequency-doubled to 515 nm (HIRO harmonics generator, Light Conversion Ltd.), and a pulse picker was used to reduce the repetition rate to 20 kHz in the single-sweep mode, ensuring a time resolution of tens to hundreds of picoseconds depending on the time window.

## Results and Discussion

### Time-Resolved Photoluminescence

**Figure 2:**
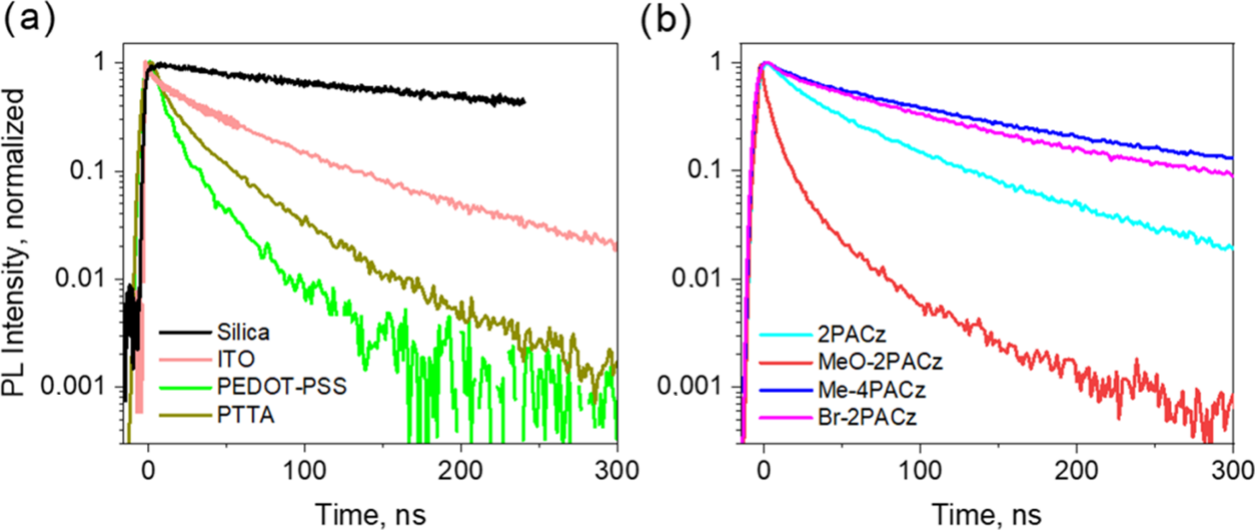
Photoluminescence kinetics of perovskite formed on silica, ITO, or polymeric hole transport layers (a) and on different self-assembling monolayers (b).

Figure 2 shows the PL decay kinetics for the perovskite devices with different hole transport layers. Perovskite deposited on a silica substrate showed almost exponential PL decay with a long lifetime, about 300 ns, which indicates the high quality of the perovskite layer. The PL decay for perovskites deposited on ITO is much faster than that observed on silica, regardless of the presence of additional transport layers. ITO with the Fermi level being inside the band gap of perovskite acts as an acceptor of both electrons and holes, thus suggesting that carrier extraction is a major PL quenching factor.^25^ It also suggests that the hole transfer at the interface, rather than carrier diffusion through the perovskite layer, predominantly limits the carrier extraction rate.^44^ All samples with hole transport layers exhibited nonexponential decays. The initial decay rates strongly varied depending on the transport layer, ranging from approximately 10 to >50 ns, revealing the different carrier extraction properties of the transport layers. Notably, both polymer hole transport layers, PEDOT:PSS and PTAA, caused very fast PL quenching, much faster than for perovskites deposited directly on ITO. This indicates that even though both holes and electrons may be extracted to ITO, the hole extraction to the investigated polymeric hole transporters is much faster. Different monolayers caused very different PL decay rates. Specifically, the MeO-2PACz monolayer caused very fast extraction, even faster than the polymer transport layers, while other monolayers somehow reduced the PL quenching rate in comparison with bare ITO. In contrast to the used polymers, the majority of the used monolayer molecules cannot accept either electrons or holes due to their large band gap and high ionization energies and thus are expected to create barriers for the carrier extraction taking place by tunneling. Consequently, they reduce the hole extraction and PL quenching rate, depending on the energy levels of monolayer molecules and their dipole moments and sizes, as will be discussed below. Monolayers may also reduce the interfacial perovskite defect states acting as nonradiative recombination centers^6^. On the other hand, MeO-2PACz has low ionization energy^28^ and therefore similar to polymeric transporters may act as a hole acceptor, causing very fast PL quenching. All investigated hole transporting materials have high energy lowest unoccupied molecular orbital (LUMO) levels^9^ and therefore create particularly large barriers for electron transfer. However, the monolayers may not completely block the electron transfer to ITO, which may occur via tunneling or through some defects in the monolayers, which are difficult to avoid. To mitigate their influence, a strategy of using additional molecules to fill the holes of SAMs was suggested by Al-Ashouri.^48^ Thermal evaporation was also demonstrated to enable more even coverage.^17,18^ Photoluminescence has limited possibilities to reveal all these processes, since the PL completely decays once one type of charge carriers are extracted, providing no information about the fate of the second type of charge carriers.

### Transient Photoresponse

#### ITO Sample

**Figure 3:**
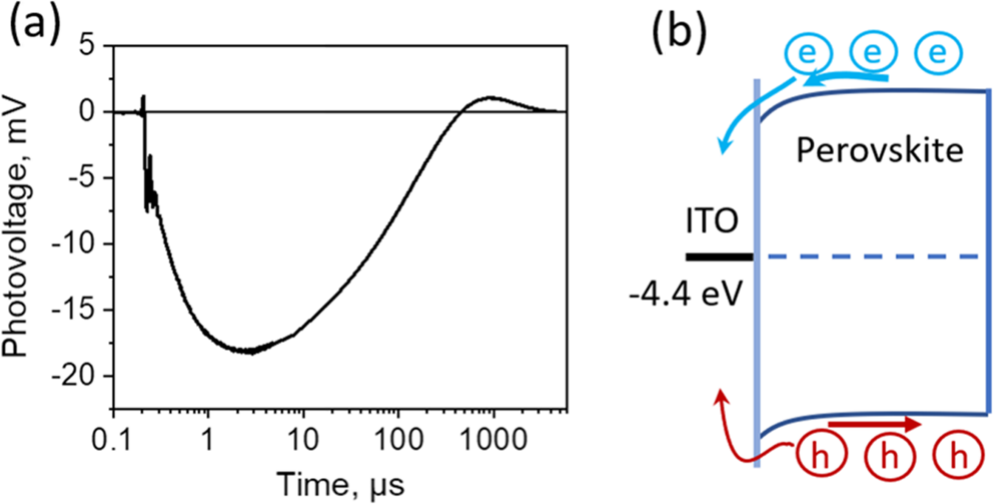
(a) Photovoltage kinetics in the ITO sample after its excitation by 532 nm, 5 ns, ∼0.1 mJ/cm^2^ energy density light pulses at a 10 Hz repetition rate. (b) Energy scheme and schematic presentation of the carrier motions in the ITO sample. Thick arrows show ultrafast carrier drift inside the perovskite layer, and thin lines show electron and hole extractions during several hundreds of microseconds.

We begin our analysis of the transient photoresponse of the samples by examining the photovoltage kinetics in the sample where perovskite was deposited directly onto the ITO layer (ITO sample). As Figure 3a shows, the ITO sample reveals a very fast initial negative voltage drop, faster than our time resolution of about 10 ns and even much faster than the photoluminescence decay. The photovoltage growth corresponds to the integrated current created by carrier drift and extraction. However, it should be noted that the voltage kinetics for the bare ITO samples varied significantly between samples. In some samples, the initial photovoltage change was even positive (see SI Figure S2). This initial fast drop can hardly be caused by the carrier extraction, which, according to PL measurements, is slightly slower. Instead, it is more likely being created by the drift of photogenerated charge carriers inside the perovskite layer caused by the internal electric field associated with the different Fermi levels of ITO and perovskite. We used the ultraviolet photoelectron spectroscopy (UPS) technique to measure the Fermi levels of both materials. UPS spectra are presented in SI Figure S3. The spectra give the Fermi level of ITO, which approximately corresponds to the work function, equal to −4.4 eV, which is in good agreement with the literature data.^47,49,50^ The Fermi level of perovskite was obtained as almost equal to that of ITO, indicating that it is slightly above the middle of the band gap, which is approximately at −4.68 eV. However, perovskite Fermi level positions apparently slightly varied from sample to sample, being slightly lower or higher than the Fermi level of ITO due to some uncontrollable variations in perovskite fabrication. It explains the variations of the photocurrent kinetics. The kinetics presented in Figure 3a may be explained by the energy diagram presented in Figure 3b, which corresponds to the perovskite Fermi level being below that of ITO. Equilibration of Fermi levels in this case causes down-bending of perovskite energy bands next to the interface with ITO.^47,49,50^ Based on this scheme, we can explain the fast negative current as created by the electron and hole drift toward and away from the interface, respectively, induced by the down-bending of energy bands. However, this fast voltage change is expected to be positive, as observed in some samples (Figure S3), in the case of the Fermi level of perovskite being above that of ITO, causing up-bending of energy levels. An additional slower voltage decrease is observed during 1–2 μs, and this decay phase agrees well with the photoluminescence decay and therefore should be attributed to electron extraction. The negative signal relaxes during tens and hundreds of microseconds and turns even slightly positive. According to the energy scheme presented in Figure 3b, the ITO electrode may extract both electrons and holes; therefore, it is reasonable to attribute the latter process to electron extraction. Finally, the positive signal decays exponentially with a time constant of about 1 ms. Taking into account a sample capacitance of about 6 nF and an oscilloscope input resistance of 1 MΩ, the sample recharging via an external measurement circuit should take place for about 6 ms. Therefore, the ∼1 ms process, which strongly varied between samples, should be attributed to sample recharging, probably accelerated by the presence of some internal shunt resistance caused by some defects of the isolating polymer layer.

#### 2PACz Sample

**Figure 4:**
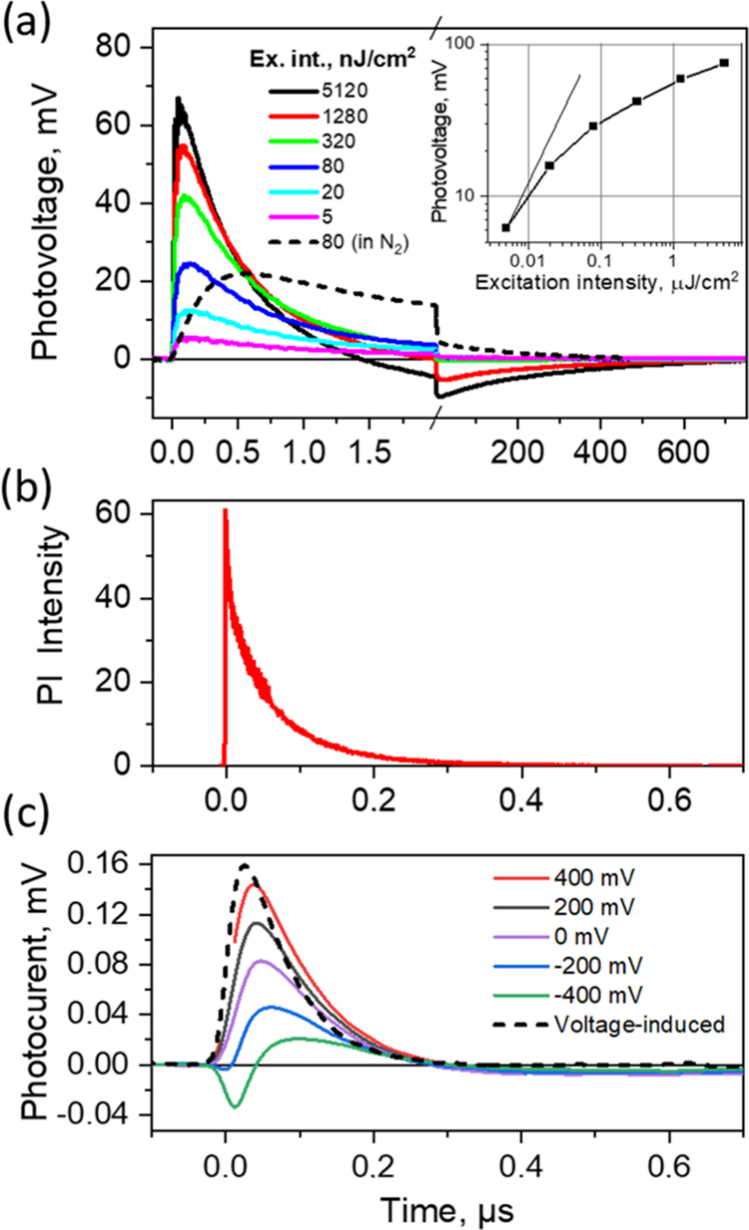
(a) Photovoltage transients for the pristine sample with the 2PACz monolayer prepared in air measured at different excitation intensities (solid lines) and for the monolayer prepared in a nitrogen atmosphere (dashed line). (b) Photoluminescence kinetics for the 2PACz sample prepared in air. (c) Photocurrent transients at 320 nJ/cm^2^ excitation intensity measured under different applied voltages. The dashed line shows a voltage-induced photocurrent transient.

Before analyzing the properties of various transport layers, we focus on the carrier dynamics in one type of sample with a 2PACz monolayer. Figure 4a shows photovoltage transients at different excitation intensities in the 2PACz sample with the monolayer prepared in air. In contrast to the ITO sample, the photovoltage kinetics of the 2PACz sample reveals a fast positive voltage growth and subsequent decay. At very low excitation intensity, the positive voltage rises for about 100 ns. This rise time agrees well with the PL decay (shown in Figure 4b for comparison). The positive current may be caused by both hole transfer to ITO and their trapping by surface states because holes in both cases move toward the ITO layer, while extraction of electrons is blocked or slowed down by the monolayer. Subsequently, the photovoltage decreases during several microseconds, suggesting that the majority of electrons also drift toward the interface and apparently recombine with holes trapped at the interface. Additionally, electrons may also be extracted from ITO because of incomplete blocking of their transfer by 2PACz.

**Figure 5:**
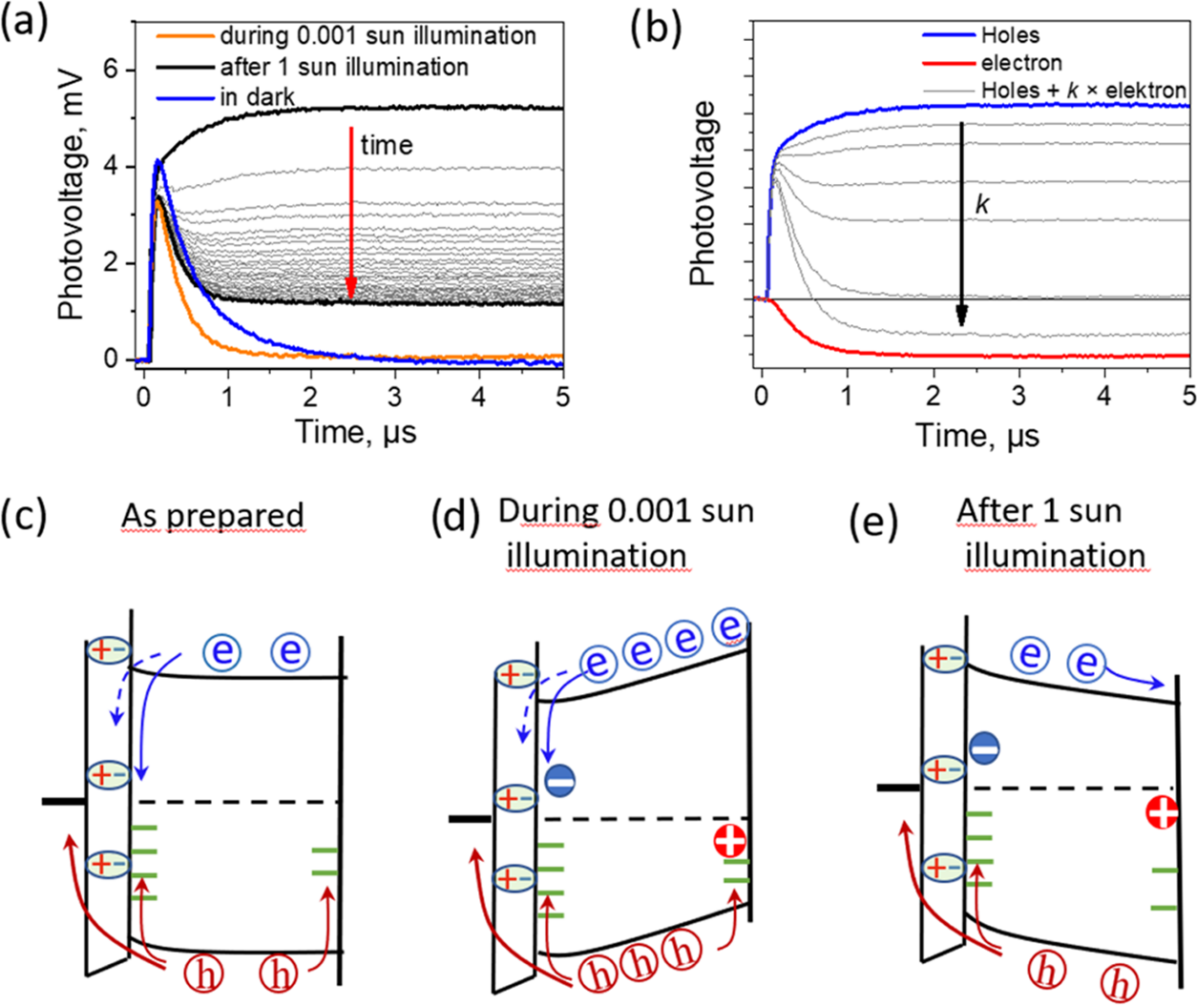
(a) Photovoltage transients measured for the 2PACz sample in the dark (blue curve), under illumination by approximately 0.001 sun white light (yellow curve), and after the sample exposure for 30 s by ∼1 sun light when measurements were performed in the dark every 10 s after the exposure was terminated (black curves). (b) Photovoltage transients modeled as a sum of invariant hole (red line) and variable electron (blue line) components. (c–e) Energy schemes of the sample before, during, and after illumination. Blue and red circles with – and + show negative and positive ions, respectively.

With increasing excitation intensity, the peak of photovoltage increases very sublinearly and almost reaches saturation at high excitation intensities (see the inset of Figure 4a). This behavior indicates that carrier recombination strongly competes with their extraction. At high excitation intensities, the photovoltage grows faster, which is most likely caused by carrier recombination, which shortens the effective carrier lifetime and, thus, also limits their extraction time. The photovoltage decay also becomes faster at higher excitation intensities. The reasons for this behavior are less obvious. We propose two possible processes: (a) Extracted holes and electrons remaining in the perovskite layer create an electric field that attracts electrons toward the interface with ITO, accelerating surface recombination. (b) Hole trap states at the perovskite/SAM interface are strongly populated at high excitation intensities, leading to subsequent recombination of electrons with the trapped holes. Remarkably, the photovoltage becomes even negative after several microseconds, which is particularly evident at high excitation intensities. The most likely explanation for this feature is that hole trap states are also formed at the perovskite interface with the insulating polycarbonate layer, and a fraction of photogenerated holes are trapped in these states and remain trapped there for a long time, while electrons remain in the perovskite bulk or are extracted to ITO. This leads to an opposite charge separation and the generation of a negative photovoltage. The negative photovoltage decays over hundreds of microseconds. This process is most likely caused by the release of the trapped holes and their extraction to ITO or their recombination with electrons remaining in the perovskite. The scheme in Figure 5c explains these processes.

Figure 4c shows the photocurrent kinetics in the 2PACz sample measured with a 50 Ω oscilloscope input resistance. Due to the weaker signal obtained in this measurement regime, the measurements were performed at a relatively high excitation intensity of 320 nJ/cm^2^. Since the isolating polymer layer forms a capacitor connected in series with the rest of the sample, by using pulsed voltage, we were able to apply an electric field to the perovskite layer in this measurement regime. At zero applied voltage, the photocurrent dynamics approximately corresponds to the derivative of the photovoltage kinetics, only measured with a lower time resolution, as discussed before. When a positive voltage is applied, the positive photocurrent becomes stronger without obviously changing the shape of the photocurrent transient. The negative voltage causes instantaneous negative current, which rapidly decays and turns into positive. Importantly, the current transients at all applied voltages may be perfectly expressed as a combination of the current transient at zero applied voltage and the voltage-induced transient obtained by subtracting kinetics at different voltages from one another, as shown in Figure 4c by the dashed line. Based on this observation, the voltage-induced photocurrent transients should be considered to be caused by the displacement of photogenerated charge carriers inside the perovskite layer driven by the applied voltage. The negative current observed in the >0.3 μs time domain is almost independent of the applied voltage, which indicates that all other processes, such as carrier extraction and recombination, are independent or are only weakly affected by the applied voltage. Consequently, the transient photocurrent data provides very limited additional information about the carrier dynamics at the interface; therefore, we will not analyze the data in the photocurrent regime for other samples.

The above-discussed photocurrent results (solid lines in Figure 4a) were obtained for the samples with a molecular monolayer prepared in air. The dashed line in Figure 4a shows the photovoltage kinetics for the samples formed on the 2PACz layer prepared in a nitrogen atmosphere. The preparation in nitrogen helps to avoid the oxidation of monolayer molecules and the adsorption of water, thereby reducing the trap density at the monolayer/perovskite interface. Indeed, the photocurrent kinetics in such samples was significantly different. The initial photovoltage growth in the sample prepared in nitrogen was much slower, taking hundreds of nanoseconds, and the subsequent photovoltage decay was also much slower compared to the sample with the monolayer prepared in air. This difference indicates that the preparation of the monolayer in air causes formation of a high density of interface trap states, which trap the majority of photogenerated holes before they are transferred to ITO. Despite hole trapping slowing down their extraction to ITO, it accelerates the growth of positive photovoltage because, as was mentioned, this process accelerates hole motion inside the perovskite layer toward the interface with ITO. Subsequently, electron recombination with trapped holes causes electron diffusion toward ITO, generating a negative current. On the other hand, much slower photovoltage growth and decay processes in the sample with the monolayer prepared in nitrogen indicate that it helps to significantly reduce the density of interface traps.

The above-discussed photocurrent results were obtained by conducting measurements in the dark (except for laser pulses) on freshly prepared samples that had not been previously exposed to light. Sample exposure to light dramatically changes the photovoltage transients. Figure 5 shows the photocurrent kinetics at a low laser pulse intensity of 5 nJ/cm^2^ for the 2PACz sample measured in the dark (blue curve) and under constant illumination by weak white light of approximately 0.001 sun intensity (yellow curve). The weak light caused a reduced photovoltage peak and faster decay of the positive photovoltage.

Next, we illuminated the sample with strong, approximately 1 sun intensity white light for 30 s and measured the photovoltage kinetics after termination of light exposure. The exposure drastically changed the photovoltage kinetics (black curves). Immediately after the exposure, the sample showed no photovoltage decay on a time scale of several microseconds; instead, the photovoltage slightly increased during this time. As the time after the sample exposure increased, the shape of the photovoltage kinetics gradually partly regained its initial shape observed before exposure. Initial transformation took place on a time scale of seconds but later slowed down, and even after 1 h, the shape of the kinetics still remained significantly different from that before the exposure. The kinetics approximately returned to the previous shape only after storage of the sample in the dark during the night (not presented).

Energy schemes presented in Figure 5 explain the sample modification caused by illumination. The molecular monolayer separating ITO and perovskite has a large energy gap and also possesses a dipole moment, which inclines energy levels, and we can consider that it increases the work function of the ITO electrode.^25^ Ionization energies and dipole moments of the investigated monolayer molecules, as well as the work functions of ITO modified by monolayers, were presented in ref (28). 2PACz molecules reduce the work function of ITO by 0.2 eV. These molecules have an ionization energy of 5.75 eV. Based on these data and measured Fermi levels of perovskite and ITO, we have constructed the energy scheme presented in Figure 5c. This scheme suggests that the monolayer creates barriers for both electron and hole extraction. However, the barrier for holes is lower, allowing them to tunnel through and be transferred to ITO. Additionally, a fraction of holes can be trapped by the interface trap states. In contrast, electron extraction is slower due to the high barrier over the monolayer. However, as already discussed, electrons can recombine with the trapped holes.

Under sample illumination by constant light, holes are extracted more efficiently than electrons. Therefore, electrons populate the conduction band, causing a shift of energy levels upward, as illustrated in Figure 5d. The additionally created internal electric field changes the motion dynamics of charge carriers generated by the laser pulse: the photogenerated holes encounter an additional barrier for their extraction, while the electrons accumulate closer to the interface and are extracted more efficiently. Consequently, the illumination causes a stronger negative current and faster decay of the positive photovoltage.

Sample illumination by the high-intensity constant light causes qualitatively similar but much stronger changes in the energy diagram, which remain modified after light termination. The observed slow processes in the exposed sample suggest that ion motion is mainly responsible for the observed dynamics. In addition to modification of the carrier dynamics, the created internal electric field causes a redistribution of the mobile ions. The negative ions accumulate at the perovskite interface with the monolayer and positive ions at the interface with the insulating polymer and partly screen the generated internal electric field. When the illumination is terminated, the energy level diagram does not return to its initial shape observed before illumination because redistributed ions create an opposite additional internal electric field directed toward the perovskite interface with the monolayer. Consequently, the energy level diagram acquires the shape shown in Figure 5e. This configuration favors hole extraction, and more importantly, it eliminates electron extraction by forcing them to move toward the perovskite interface with the insulating polymer. As a result, photocurrent kinetics shows only the positive current created by the hole extraction and by diffusion of electrons away from the perovskite/monolayer interface. Additionally, trapped holes gradually jump out of the traps and transfer to ITO, causing an additional positive current component observed for a few microseconds. Over time, after the sample has been illuminated, the ions gradually diffuse back to their initial distribution, and the photocurrent kinetics also acquires its initial shape observed before the sample illumination. This process is widely extended in time: initially, it takes place on a time scale of a few tens of seconds. Later, it slows down. As mentioned, we can observe the effect of the sample illumination even after about an hour, and finally, the sample returns to its initial condition only overnight. Such slow processes are quite common in perovskites. For example, we have previously observed electric field-induced photoluminescence quenching dynamics taking place on a time scale of seconds, minutes, and hours and have also attributed it to the motion of ions.^51,52^

We can express the photocurrent kinetics before illumination and at different times after illumination by different contributions of positive and negative components shown in Figure 5b, which should be attributed to the cumulative hole and electron currents. The negative electron current related to their recombination with the trapped holes starts with some delay, apparently caused by the delayed population of the hole trap states, and proceeds slightly slower, in agreement with the photovoltage decomposition. We can model the photocurrent kinetics under different illumination conditions by combining hole and electron currents with different contributions. As Figure 5b shows, we obtain a perfect agreement between the experimental and modeled curves assuming that the cumulative hole current is independent of the sample illumination, while the cumulative electron current changes from being negligibly observed immediately after termination of illumination and becomes comparable to the cumulative hole current long time after the illumination when the redistributed ions return to their initial positions.

#### Samples with Different Hole Transport Layers

**Figure 6:**
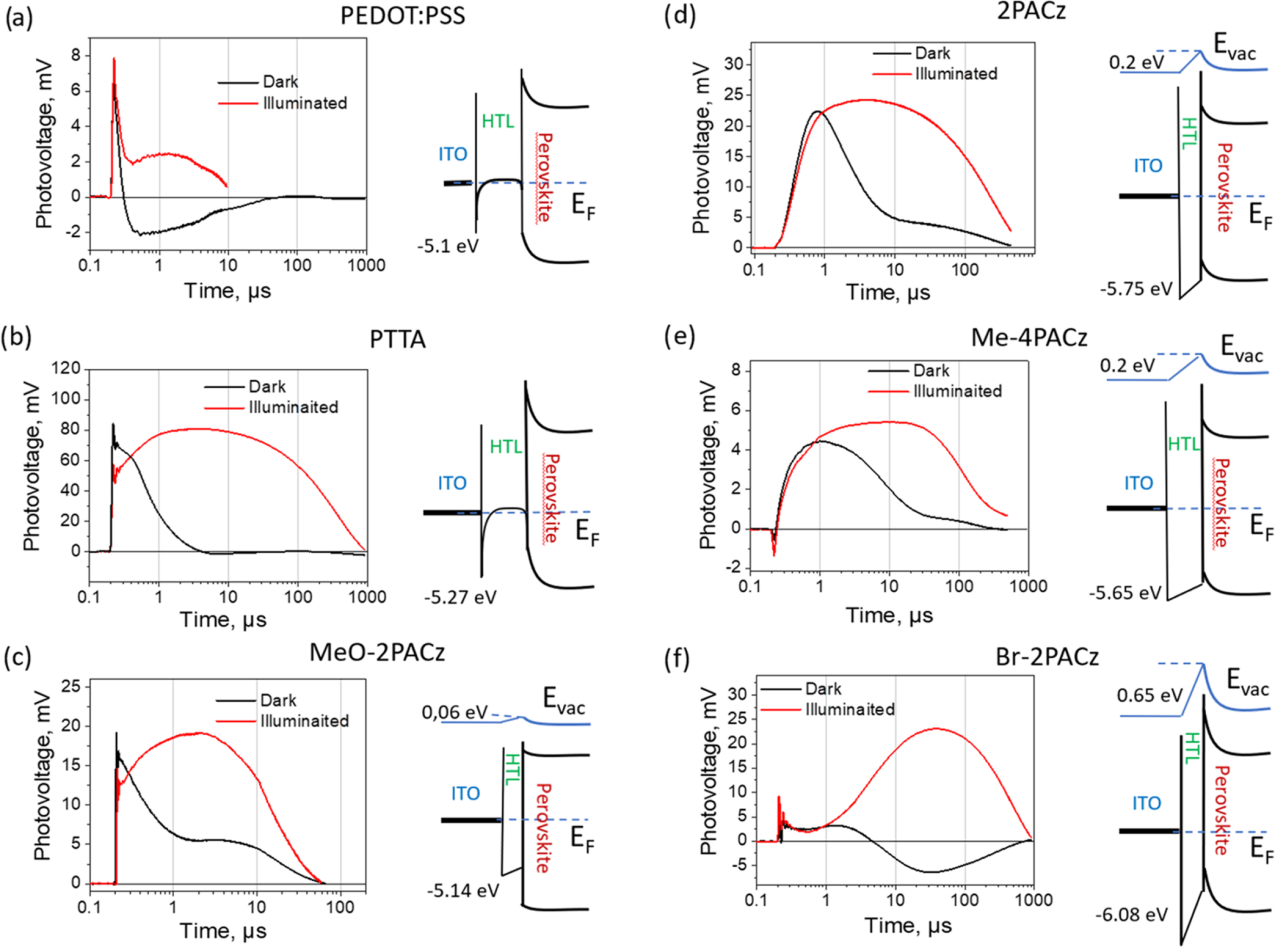
Photovoltage transients measured at ∼10 nJ/cm^2^ excitation intensity for samples with polymeric (a, b) and SAM (c–f) transport layers. Black lines show transients for non-exposed to constant light samples, and red lines show transients obtained immediately after the sample illumination for 30 s by ∼1 sun light. Images on the right side show energy diagrams evaluated by accounting energy levels of ITO, perovskites, and hole transporting materials. The numbers in energy diagrams indicate ionization energies of hole transporting materials (bottom numbers) and the increase of vacuum energies (as well as the increase of apparent ITO work function) caused by the dipole moments of SAMs (top numbers) evaluated from the data of ref (30). Equilibration of Fermi levels causes up-bending of perovskite energy bands.

The data presented above show how strongly the carrier dynamics depends on the sample preparation and exploitation conditions. This strong dependence complicates the comparison of the carrier dynamics between samples with different hole transport layers. To minimize this influence, we attempted to maintain identical sample preparation and investigation conditions and fabricated several samples of each type to ensure that the results were repeatable. Figure 6 shows the most typical photocurrent kinetics measured in several samples of each type in the dark and after their exposure to 1 sun light. Kinetics for more samples are presented in SI Figure S4. The energy schemes presented on the right side are designed as described above, accounting for the equilibration of Fermi levels on the basis of energy parameters of monolayer molecules presented in ref (28).

Photovoltage kinetics strongly depends on the type of HTM used. Samples with polymer HTMs and those utilizing the MeO-2PACz monolayer demonstrate a very fast initial positive voltage jump. This fast voltage growth aligns with the fastest photoluminescence decay observed in these samples. The polymer transport layers are degenerate p-type semiconductors with Fermi levels positioned inside the valence band. Equilibration of the Fermi levels of ITO, HTMs, and perovskites causes formation of Schottky barriers and bending of energy bands of perovskites, as shown in energy diagrams, facilitating hole extraction. MeO-2PACz, possessing low ionization energy, also shows very fast hole extraction, in agreement with the energy diagram, suggesting no barrier for hole extraction. Transport layers of all these samples, including the MeO-2PACz monolayer, have high energy highest occupied molecular orbital (HOMO) levels and may accommodate holes. However, extracted holes experience Schottky barriers for further extraction to ITO and remain in transport layers in close contact with perovskite and therefore may easily recombine with the electrons remaining in perovskites, probably involving interface states, causing very fast photovoltage decay in the dark, in some cases even to negative values. In the case of PEDOT:PSS, the voltage drops to a negative value. Apparently, a high density of electron traps is formed in the sample, which trap electrons, leaving a fraction of photogenerated holes in a bulk of the perovskite layer and, thus, causing a negative balance of holes and electrons trapped by the interface or transferred to transport layers and electrodes.

Sample exposure to strong light weakly affects the very initial hole transfer processes. However, at longer times, we observe additional growth lasting for several microseconds instead of the voltage decay observed before exposure. This behavior is attributed to the internal electric field created by redistributed ions that hinder electron motion toward the ITO electrode. This relatively slow voltage growth should be attributed to the holes and/or electrons trapped in the bulk of the perovskite layer, which are driven by the internal electric field to create positive voltage. The final voltage decay during hundreds of microseconds is caused by the sample discharging through the external circuit.

Samples with other monolayers show a much slower voltage growth. Molecules such as 2PACz, Me-4PACz, and Br-2PACz have lower HOMO level energies, resulting in barriers for hole extraction. Therefore, the hole extraction and, thus, the voltage growth are significantly slower. These molecules also have large dipole moments, reducing ITO work function and causing strong up-bending of perovskite energy levels. Such band bending favors hole accumulation close to the interface and probably their trapping. This particular energy diagram is apparently responsible for the voltage kinetics with two peaks that indicate hole extraction occurring on time scales of submicroseconds and tens of microseconds in samples exposed to strong light. The fast voltage peak reveals the extraction of holes before they are trapped in the interface trap states, while the slow component reveals the extraction of thermally released holes. This behavior is particularly clear in the case of the Br-2PACz sample. Before illumination, this sample shows weak ultrafast voltage growth and almost no slower second extraction phase. The fast growth component is much faster than the relatively slow photoluminescence decay (Figure 2). Therefore, this fast photovoltage jump should be attributed to the drift of holes within the perovskite layer caused by the strong band bending rather than to hole extraction. Hole extraction across the high barrier occurs only after sample illumination and proceeds very slowly over about 100 μs. The low HOMO levels of 2PACz, Me-4PACz, and Br-2PACz rule out the population of these monolayers with holes. The up-bending of energy levels in the perovskite layers of these samples also causes the repulsion of electrons away from the interface. Consequently, electrons and holes are spatially separated and cannot recombine. It causes much slower carrier recombination and slower photovoltage decay for pristine non-exposed samples than for the samples shown on the left side of Figure 6.

As the data presented in Figure 6 show, the charge carrier dynamics at the interface between the hole transport layer and perovskite strongly depends on the transport layers, particularly for pristine, non-exposed-to-light samples. On the other hand, the internal electric field created by redistributed ions separates charge carriers and prevents their recombination, making photovoltage differences between the samples less significant. Therefore, although photovoltage investigations reveal significant differences between different transport layers, evaluation of their performance in operating solar cells is not straightforward, as it may vary depending on the perovskite composition and fabrication procedures as well as on solar cell exploitation conditions.

## Conclusions

Using time-resolved photovoltage measurements in an incomplete p–i–n perovskite solar cell with blocked electron extraction, we have investigated hole extraction and electron–hole recombination dynamics at the interface between the perovskite and the hole transporting layer. Carrier extraction and recombination dynamics were found to depend significantly on the properties of both the transport layers and perovskite. We observed a clear dependence of the carrier extraction dynamics on the ionization energies and dipole moments of SAMs. Molecules with large ionization energies create barriers that retard hole extraction. On the other hand, monolayers consisting of molecules with large dipole moments reduce the work function of ITO, resulting in the upward bending of the perovskite energy levels and repulsion of electrons from the interface, which facilitates effective separation of electrons and holes and plays a crucial role in preventing interface recombination. Sample illumination by constant light significantly changes the perovskite properties. Illumination by weak light populates the perovskite layer with electrons and accelerates interface electron–hole recombination. In contrast, short-term exposure to strong light creates residual changes in the perovskite properties related to the redistribution of ions, which last for minutes to hours and strongly affect the carrier dynamics. The clarified mechanisms of charge transfer and recombination at the interface between perovskite and hole transport layers will improve our understanding of the relationships between the properties and functions of the transport layers, essential for the further development of perovskite solar cells

## Supporting Information Available

SEM image; UPS spectrum; and transient photovoltage kinetics (PDF)
