# Supplementary material for: Charge Carrier Dynamics at the Perovskite Interface with Self-Assembled Monolayers
Source: ACS Appl Mater Interfaces. 2024 Oct 18;16(43):59477–87. doi: 10.1021/acsami.4c10223 (PMC11533155; doi:10.1021/acsami.4c10223)
Supplement: Supplementary file 1 — am4c10223_si_001.pdf [file am4c10223_si_001.pdf]

Supporting Information

Charge Carrier Dynamics at the Perovskite Interface with Self-Assembled Monolayers.

Ernestas Kasparavičius, Marius Franckevičius, Simonas Driukas, and Vidmantas Gulbinas,*

Center for Physical Sciences and Technology, Saulėtekio av.3, Vilnius, 10257, Lithuania

e-mail: ernestas.kasparavicius@ftmc.lt

KEYWORDS: perovskite, self-assembling monolayers, hole transport layer, photocurrent dynamics, photovoltage transients, ion redistribution.

a) ITO

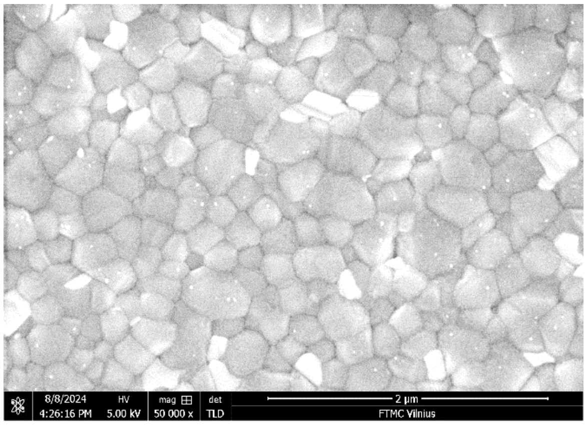

b) PTTA

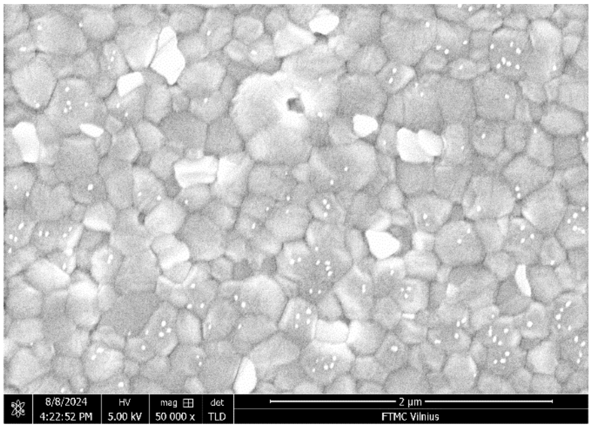

c) PEDOT:PSS

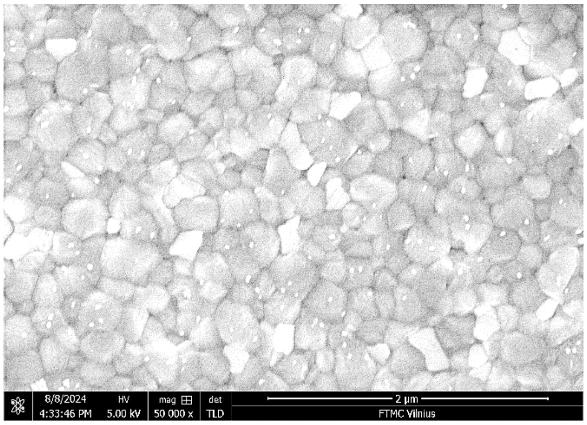

d) 2PACz

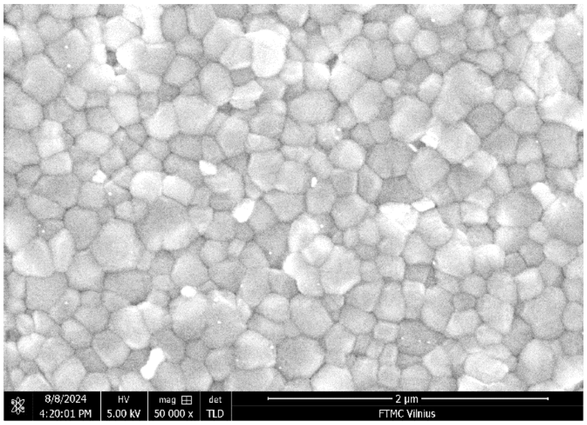

e) MeO- 2PACz

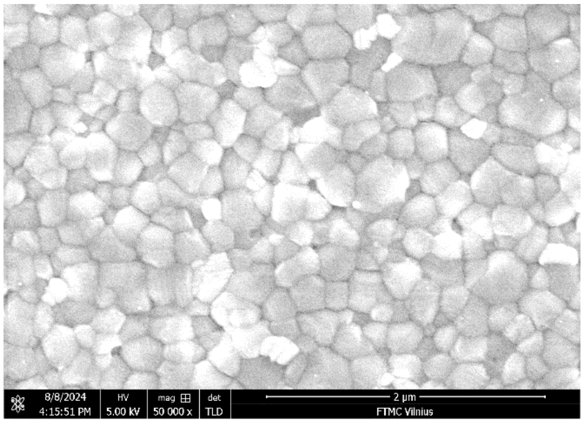

f) Me-4PACz

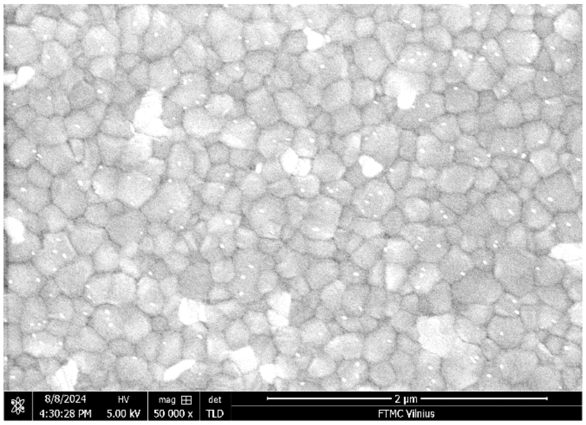

Figure S1. Surface scanning electron microscope images of perovskite with different HTM layers: a) ITO; b)PTTA; c) PEDOT:PSS; d) 2PACz; e)MeO-2PACz; f)Me-4PACz.

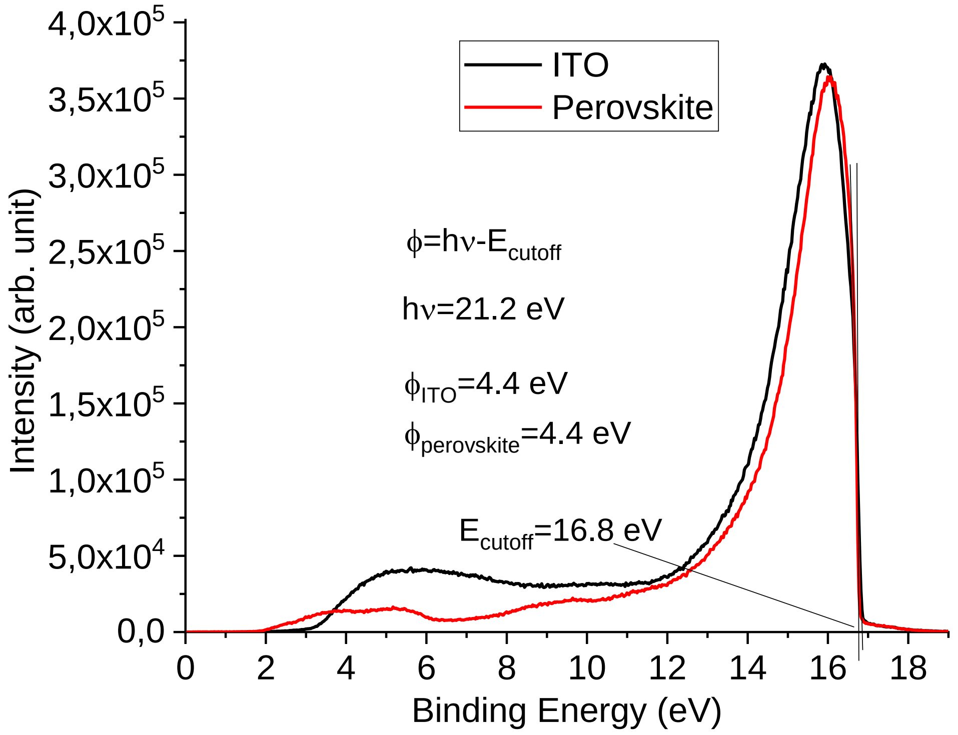

Figure S2. ITO and perovskite ultraviolet photoelectron spectroscopy (UPS) spectrum. Measured by XPS-UPS spectrometer AXIS Supra+

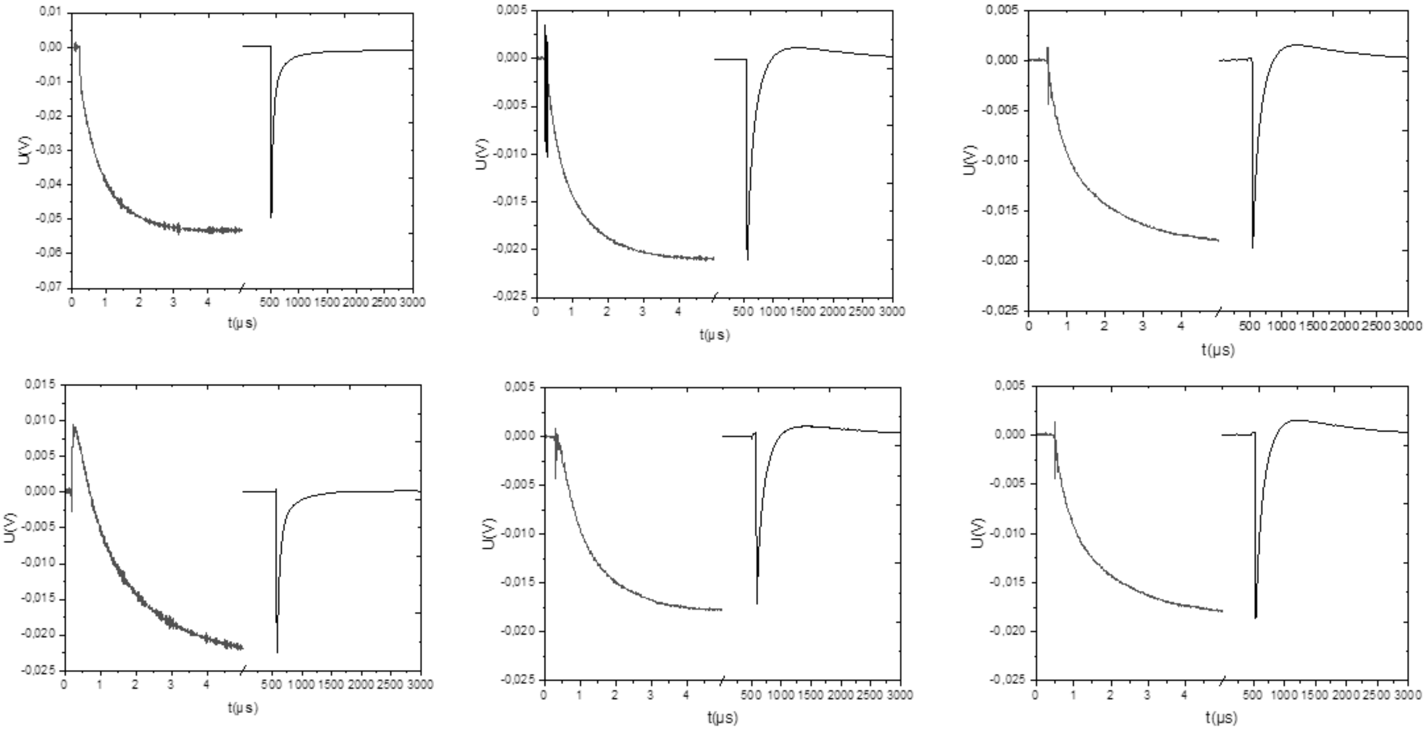

Figure S3. Transient photovoltage kinetics in samples with perovskite formed directly on ITO

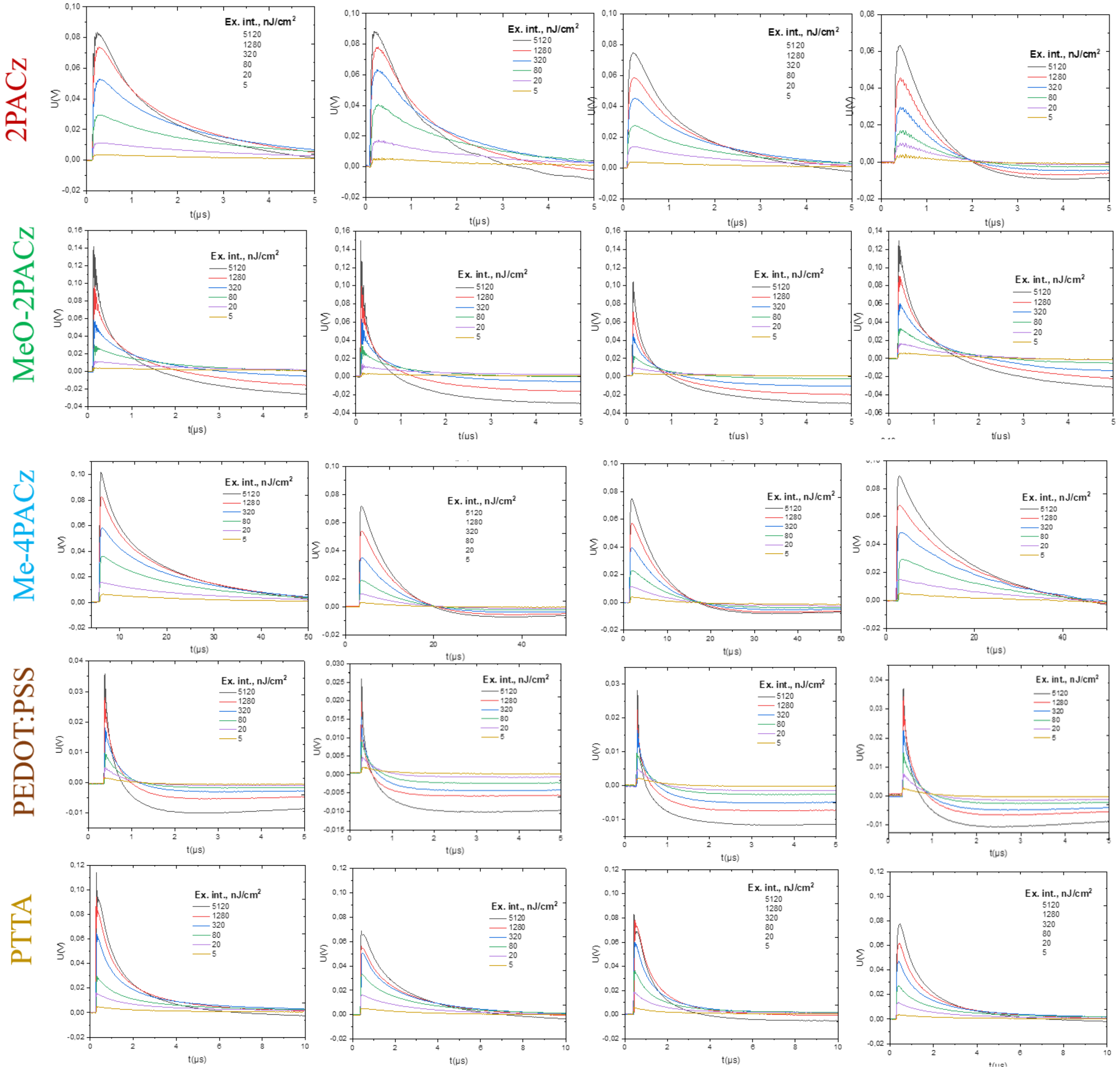

Figure S4. Transient photovoltage kinetics for samples with different transport layers at different excitation intensities.
